# Characterization and Applications of Metal Ferrite Nanocomposites

**DOI:** 10.3390/nano12010107

**Published:** 2021-12-30

**Authors:** Thomas Dippong

**Affiliations:** Faculty of Science, Technical University of Cluj-Napoca, 76 Victoriei Street, 430122 Baia Mare, Romania; thomas.dippong@cunbm.utcluj.ro

## Introduction

In recent years, nanosized spinel-type ferrites emerged as an important class of nanomaterials due to their high electrical resistivity, low eddy current loss, structural stability, large permeability at high frequency, high coercivity, high cubic magnetocrystalline anisotropy, good mechanical hardness, and chemical stability. Thus, research dedicated to the development and characterization of such nanomaterials, the development of cost-effective, eco-friendly synthesis methods, and finding new applications for existing materials has received considerable attention. Metal ferrites MFe_2_O_4_ (M = Mn^2+^, Co^2+^, Ni^2+^, Mg^2+^, Zn^2+^) have been promoted as a novel group of versatile nanomaterials due to their tunable magnetic, electrical and optical properties together with good thermal and chemical stability. They are suitable for a wide range of potential applications in photoluminescence, catalysis, photocatalysis, humidity-sensors, gas sensors, biosensors, information storage, permanent magnets, transformer cores, radiofrequency circuits, waveguide isolators, hybrid supercapacitors, ferrofluids, inductors, converters, antennas, biocompatible magnetic-fluids, magnetic drug delivery, magnetic refrigeration, microwave absorbers, water decontamination and medical imaging, ceramics pigment, corrosion protection, antimicrobial agents, and biomedicine (hyperthermia) [1,2,3,4,5,6,7].

This Special Issue focuses on ferrite-based nanomaterial synthesis and characterization including (i) synthesis; (ii) advanced chemical and physical characterization of structure and properties; (iii) magnetic behavior; (iv) computational and theoretical studies of reaction mechanisms, kinetics, and thermodynamics; (v) applications of nanomaterials in environmental, biological, catalytic, medical, cultural heritage, food, geochemical, polymer, and materials science.

The control of the morphology and magnetic properties of ferrite nanoparticles (NPs) is critical for the synthesis of compatible materials for different applications [8,9]. In this Special Issue, Duong et al. [10] reported the synthesis of CoFe_2_O_4_ NPs by a solvothermal method using cobalt nitrate and iron nitrate as precursors, and oleic acid as a surfactant. Additionally, the effect of reaction time, reaction temperature, and oleic acid concentration on the properties of CoFe_2_O_4_ nanoparticles was investigated. The obtained results indicated that the oleic acid concentration plays an important role in controlling the morphology and properties of the CoFe_2_O_4_ NPs. The obtained high-quality CoFe_2_O_4_ NPs with improved magnetic performance are a potential candidate for many applications, such as bio-separation, magnetic resonance imaging, biosensors, drug delivery, and magnetic hyperthermia [10].

Due to its excellent stability, non-toxicity, biocompatibility, drug loading capacity, and water dispersibility, the mesoporous SiO_2_ enhances the stability of NPs in water, improves chemical stability and minimizes the agglomeration of NPs, without influencing their magnetic and dielectric properties [1,8,9]. In this Special Issue, the effect of SiO_2_ embedding on the production of single-phase ferrites, as well as on the structure, morphology and magnetic properties of (Zn_0.6_Mn_0.4_Fe_2_O_4_)_δ_(SiO_2_)_100−δ_ (δ = 0–100%) NPs synthesized by the sol–gel method and annealed at different temperatures was reported by Dippong et al. [2]. The obtained results indicated that the preparation route strongly influences the particle sizes, and especially the magnetic behavior of the NPs. The obtained average crystallite size was 5.3–27.0 nm at 400 °C, 13.7–31.1 nm at 700 °C and 33.4–49.1 nm at 1100 °C, respectively. The Zn_0.6_Mn_0.4_Fe_2_O_4_ embedded in SiO_2_ exhibited superparamagnetic-like behavior, whereas the unembedded Zn_0.6_Mn_0.4_Fe_2_O_4_ behaved like a high-quality ferrimagnet [2].

This Special Issue also includes the study of Dumitru et al. [11] on Bi_2_Cu(C_2_O_4_)_4_·0.25H_2_O synthesis by thermolysis, followed by its integration within a CuBi/carbon nanofiber (CNF) paste electrode and its application in the electrochemical detection of amoxicillin (AMX) in aqueous solution. The obtained results indicated the potential use of a CuBi/CNF paste electrode for AMX detection in aqueous solutions. By adding a concentration step into the detection protocol, the selective and simultaneous detection of AMX in a multi-component matrix is also possible [11].

I am grateful to all the authors for their contributions, covering the most recent progress and new developments in the field of metal–ferrite nanocomposites, and hope that the published studies will pave the way for novel real-world applications of green nanomaterials.

## Data Availability

Not applicable.
